# Assessing the Applicability of the GTR Nucleotide Substitution Model Through Simulations

**Published:** 2007-02-04

**Authors:** Laurent Gatto, Daniele Catanzaro, Michel C. Milinkovitch

**Affiliations:** Laboratory of Evolutionary Genetics, Institute for Molecular Biology and Medicine, Université Libre de Bruxelles, CP300, rue Jeener et Brachet 12, 6041 Gosselies, Belgium

**Keywords:** GTR model, simulations, nucleotide substitution, homogeneity, phylogeny inference

## Abstract

The General Time Reversible (GTR) model of nucleotide substitution is at the core of many distance-based and character-based phylogeny inference methods. The procedure described by Waddell and Steel (1997), for estimating distances and instantaneous substitution rate matrices, **R**, under the GTR model, is known to be inapplicable under some conditions, *ie*, it leads to the inapplicability of the GTR model. Here, we simulate the evolution of DNA sequences along 12 trees characterized by different combinations of tree length, (non-)homogeneity of the substitution rate matrix **R**, and sequence length. We then evaluate both the frequency of the GTR model inapplicability for estimating distances and the accuracy of inferred alignments. Our results indicate that, inapplicability of the Waddel and Steel’s procedure can be considered a real practical issue, and illustrate that the probability of this inapplicability is a function of substitution rates and sequence length.

We also discuss the implications of our results on the current implementations of maximum likelihood and Bayesian methods.

## Introduction

All phylogeny inference methods are based on explicit or implicit assumptions whose validity can possibly be challenged when analysing real data. Molecular genetic markers (mostly DNA sequences) have arguably become the most popular and powerful source of data for phylogeny inference. Many methods for reconstructing trees from DNA sequences (eg, distance-matrix methods, Maximum Likelihood and Bayesian approaches) rely on a substitution model that describes how sequences evolve over time. Different models, ranging from the JC model (Jukes and Cantor 1969) (assuming equal nucleotide frequencies and identical substitution rates) to the General Time Reversible (GTR) model (Lanave et al. 1984), (allowing for different nucleotide frequencies and 6 different substitution rates) have been developed.

The GTR model is a stationary Markov process by which substitution probabilities among nucleotides are expressed in the form of a matrix **P**(*t*). The GTR model assumes that the equilibrium character state frequencies and the instantaneous transition probabilities remain constant through time. The dynamic of substitution probabilities for an infinitesimal time *dt* is described by

(1) P(t+dt)=P(t) (I+Rdt)

where **I** and **R** are four-by-four real matrices representing, respectively, the identity matrix and the *instantaneous substitution rate* matrix (*ie*, the instantaneous substitution probabilities among the four nucleotides).

Lanave et al. (1984) and Rodriguez et al. (1990) have shown that

(2) $$P(t)=e^{R\text{t}}$$

is a solution of equation (1) such that, once **R** is given, the probability **P**(*t*) of substitution between two states can be computed for any *t* and the evolution of sequences through time is completely described as long as all GTR-assumptions are verified.

The transition rate matrix **R** is generally unknown and many inference methods rely on its computation: (*i*) distance methods evaluate the GTR distance *t̂* for each sequence pair and require that the corresponding **R** (see below) is Markovian (*ie*, is a real matrix with negative diagonal elements and non-negative elements outside the diagonal), and (*ii*) Maximum Likelihood and Bayesian methods require estimating **R** for computing **P**(*t*) that, in turn, is required for computing the Likelihood of a tree (Felsenstein 2004). In most phylogeny inference packages (eg, PAUP* (Swofford 2003) and MrBayes (Ronquist and Huelsenbeck 2003)), homogeneity across the tree is assumed, *ie*, a single **R** matrix is optimized for the whole tree.

On the basis of the seminal work by Lanave et al. (1984) and Rodriguez et al. (1990), Waddell and Steel (1997) proposed an exact estimation procedure to compute GTR distances (also implemented in PAUP* (Swofford 2003)). For any pair of sequences, the GTR distance is defined as

(3) $$\hat{t}=-trace[\Pi \log(P)]$$

where **log**(**P**) is the logarithmic matrix function of the net transition matrix **P**. In turn, **P** can be computed using

(4) $$P=\Pi^{-1}F^{\#}$$

where Π is the diagonal matrix whose elements are the nucleotide frequencies at equilibrium (eg, estimated from the corresponding pairwise alignment) and **F**^#^ is the *symmetrized* form of the *divergence* matrix **F** (computed from the corresponding pairwise alignment). **Log**(**P**) can then be evaluated via diagonalization: *ie*,

(5) $$\log(P)=\Omega \log[\Lambda ]\Omega^{-1}$$

where **Ω** and **Λ** are the eigenvector matrix and the eigenvalue diagonal matrix of **P**, respectively. Finally, the rate matrices **R** (for each sequence pairs) can be evaluated using

(6) $$R=\frac{\log(P)}{\hat{t}}$$

As noted by (Rodriguez et al. 1990, Waddell and Steel 1997, Yang and Kumar 1996), if at least one of the four eigenvalues of **P** is negative, the logarithmic matrix function computed by equation (5) is not defined. If applied, the procedure would contradict the Markovian hypothesis underlying the GTR model and lead to the presence of complex numbers as transition rates (which has, to our knowledge, no biological meaning).

In the framework of ML phylogeny inference from multiple sequence alignments, Yang and Kumar (Yang and Kumar 1996) proposed to use a mean **F**^#^ matrix (*ie*, the average of all **F**^#^ matrices, each computed from the corresponding pairwise sequence comparison) for computing a single **R** for the whole tree. This procedure reduces, but does not eliminate, the risk of computing a complex **R**^1^. On the other hand, many phylogeny inference softwares implement optimization techniques that yield a single **R** for the whole tree. This approach removes the possibility of observing negative eigenvalues in **P** (because computation of **log**(**P**) is by-passed) but sacrifices the possibility of locally optimizing the transition rates (eg, for each pair of nodes) and thus constrains the hypothesis of homogeneity along the whole evolutionary tree (an assumption that can be unreasonable with some data sets). When locally computing a **R** matrix (eg, for a pair of sequences) using the procedure of Waddell and Steel (1997), the homogeneity assumption only holds for the corresponding portion of the tree.

Recently, Catanzaro et al. (2006) have formally characterized the mathematical conditions, (and discuss their biological interpretation) that lead to the inapplicability of the GTR model, investigated, from a mathematical point of view, the relations between the occurrence of negative eigenvalues and both sequence length and sequence divergence, proposed a possible procedure (CPM) for estimating **R** in terms of a non-linear optimization problem (that can be implemented without assuming homogeneity across the tree), and analyzed the goodness of this new approach. However, this work was purely theoretical and did not asses whether negative eigenvalues would occur in biologically-realistic situations using the GTR model. Here, we particularly investigate whether negative eigenvalues occur under circumstances where divergence among sequences is sufficiently low not to cause major multiple alignment problems.

## Approach

Although we will focus, throughout the present paper, on GTR distances, the problems discussed below can be relevant for computing **R** matrices under a ML or Bayesian framework. For evaluating whether the inapplicability of the GTR distance estimation of Waddell and Steel (1997) is a practical issue, (*i*) we simulated, along a tree topology, the evolution of DNA sequences under the GTR nucleotide substitution model using a set of given, biologically realistic, **R** matrices in the presence or absence of insertions and deletions; (*ii*) we analyse the accuracy of simulated dataset alignments using classical methods; (*iii*) we compute the frequency of occurrence of **P** matrices characterized by negative eigenvalues; and (*iv*) we investigate the relations between, on one hand, the probability of observing negative eigenvalues of **P** and, on the other hand, evolutionary divergence among sequences, length of sequences, and deviation from the homogeneity hypothesis. As it is clear that probability of the GTR model inapplicability, but also of alignment inference inaccuracy, increase with divergence among sequences (Catanzaro et al. 2006), we performed estimation of the alignment accuracy (point (*ii*) above) as a benchmark. Indeed, as alignment of sequences is a prerequisite to meaningful phylogeny inference, we consider that any analytical problem (here, the occurrence of negative eigenvalues) arising only for sequences that are too divergent to be aligned with accuracy, is unlikely to be a practical issue. Our analyses improve understanding of the conditions of inapplicability of the GTR estimation and hints at the necessity of implementing alternative algorithms and models to deal with this issue.

### Methods

**Figure 1:**
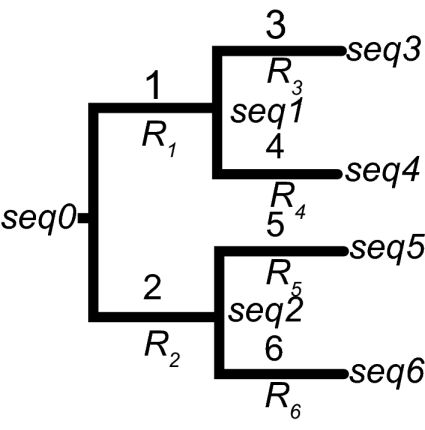
Tree topology along which the sequences have been simulated. Four different tree lengths have been analyzed. The trees are described by giving the length of branches 1 to 6: tree *T*0 = {1, 1, 2, 2, 2, 2}; tree *T*1 = {2, 2, 4, 4, 4, 4}; tree *T*2 = {2, 2, 8, 4, 8, 4} and tree *T*3 = {5, 4, 8, 12, 10, 15}.

All simulations were performed along a single symmetric topology leading to four terminal taxa (*seq3, seq4, seq5* and *seq6* on figure 1); four different sets of branch lengths (figure 1) were used. Trees *T0* to *T3* have total lengths (*ie*, the sum of the branch lengths) of, respectively, 10, 20, 28, and 54 units. Each branch is associated to a **R** matrix (see figure 1). Three situations (S1, S2, and S3) have been analyzed: (S1) All six branches are associated to the same single rate matrix (whose elements are taken from real data (Waddell and Steel 1997)):

$$R_{1..6}=\left(\begin{array}{rrrr} -0.0524 & 0.0042 & 0.0466 & 0.0016\\ 0.0042 & -0.1008 & 0.0002 & 0.0963\\ 0.1078 & 0.0006 & -0.1091 & 0.0007\\ 0.0002 & 0.1154 & 0.0007 & -0.1176 \end{array}\right)$$

(S2) Matrices *R*_1_ to *R*_5_ are as in S1, whereas matrix ***R***_6_ is as follows:

$$R_{6}=\left(\begin{array}{rrrr} -0.0501 & 0.0033 & 0.0453 & 0.0016\\ 0.0041 & -0.1008 & 0.0004 & 0.0963\\ 0.1078 & 0.0006 & -0.1100 & 0.0016\\ 0.0064 & 0.0990 & 0.0056 & -0.1110 \end{array}\right)$$

(S3) Matrices *R*_1_ to *R*_5_ are as in S1, whereas matrix ***R***_6_ is modified as follows: lines 2 and 3 have been swapped, while the second and third cells within each of these two lines have been exchanged to maintain the validity of the matrix, *ie,* the occurrence of negative diagonal elements and sums of rows = 0:

$$R_{6}=\left(\begin{array}{rrrr} -0.0524 & 0.0042 & 0.0466 & 0.0016\\ 0.1078 & -0.1091 & 0.0006 & 0.0007\\ 0.0042 & 0.0002 & -0.1008 & 0.0963\\ 0.0002 & 0.1154 & 0.0007 & -0.1176 \end{array}\right)$$

The first situation (S1) corresponds to the classical implementation of the GTR model, *ie*, homogeneity of *R* across the tree. As there is no reason to consider that, with real data, all branches of a tree must be characterized by the same rate matrix, situations (S2 and S3) might be biologically more realistic. To evaluate the differential impact (DI) on sequence evolution of two matrices A and B, we use the formula *DI* = ∑||*A_ii_*| − |*B_ii_*||. DI is (− 0.0023, 0, 0.0009, − 0.0066) for S2 and (0, 0.0083, − 0.0083, 0) for S3. In other words, in the S2 situation, the sequence experiencing a shift in rate matrix (at the base of branch 6) will instantaneously start to loose 0.0023 more A’s, 0.0066 more T’s, and 0.0009 less G’s (whereas the rates of gains/losses of C’s will remain unchanged) per unit of time. By summing the absolute values of the elements of DI, we quantify the difference of absolute overall amount of divergence that these matrices will induce to evolving sequences: *ie,* 0.0098 and 0.0166 for S2 and S3, respectively.

We also performed all simulations using two different lengths for the root sequence (*seq0* on figure 1): 200 and 1000 base pairs. To investigate the effect of sampling, we performed a third set of analyses with 200 base pairs extracted from the simulations of 1000 base-pair-long strings. In all cases, we used an initial frequency of 0.25 for each nucleotide state in the root sequence. The unit of time parameter was set to 0.01 for the 200 base-long root sequence simulations and to 0.002 for the 1000 base-long root sequence simulations. Simulations were iterated 100 times under each of the 12 conditions described above.

### Simulations without indels

The procedure that induces substitutions is at the core of our simulations: it simulates the stochastic process responsible for the sequence evolution assuming the neutrality hypothesis (Kimura 1968). Let’s consider a base *x* of the sequence *S* at position *i* at time *t*_0_, and let’s represent the possible four states {*A, C, G, T*} that *x* can take at time *t* as a pie chart equally divided. Each quarter is associated with a transition probability *p_x j_*, where *j*∈{*A, C, G, T*}. By starting from a randomly chosen quarter *j*, the final quarter (*ie*, the state to which the initial base will be substituted) is chosen by adding the transition probabilities *p_x j_* until the sum is greater than or equal to a uniformly distributed pseudorandom real number *r* in [0,1] (see (Dorigo and Stützle 2003) for details about the algorithm).

When the simulations are performed without implementing an insertion/deletion process, the correct alignments are immediately obtained from the tip sequences. These alignments are used as reference against which ClustalW-generated alignments (Thompson et al. 1994) are compared.

### Simulations with indels

To implement the insertion/deletion process, we incorporated the following parameters. The maximum number of insertion/deletion events is randomly chosen between 0 and one third of the branch length. The nature of the event, *ie*, whether it will be an insertion or a deletion, depends on the insertion/deletion ratio, here set to 1/3.5 (Zhang and Gerstein 2003). The size of an insertion/deletion is chosen from a power-law function *f_k_* = *a* × *k*^−^*^b^* describing the probability of having a gap of length *k*. Note that we limited the sum of lengths of all insertion/deletion events to be, on each branch, ≤ 5% of the sequence length at the corresponding parent node (to avoid too many gaps, hence, major alignment problems). Two different functions have been used for insertions and deletions with parameter values *a_ins_* = 0.53, *b_ins_* = 1.6 and *a_del_* = 0.48, *b_del_* = 1.51. See Zhang and Gerstein (Zhang and Gerstein 2003) for a discussion about power-law function parameter values. When the procedure inducing insertion/deletion is called, a base at position *i* in the sequence *S* is randomly chosen and used as starting point for the insertion/deletion process. The number of insertion(s) on the sequence *S* is computed by the formula

$$Round\left[\frac{MaxNumberOfIndels}{1+idratio}\right]$$

while the number of deletion is computed according to

$$Round\left[idratio\times Round\left[\frac{MaxNumberOfIndels}{1+idratio}\right]\right]$$

where *Max Number Of Indels* is the the maximum number of insertion/deletion events as defined above. The number of bases to be deleted or inserted is chosen according the power-law. Each base of an inserted block is chosen by calling the procedure introducing mutations (see above) using as input the base at position *i*.

During the simulations, each insertion/deletion event is recorded. These events are subsequently remapped and accordingly propagated into the tip sequences (removal of one or more bases in the child sequences in case of a deletion, or addition of one or more bases in the child sequences in case of an insertion) to recover the correct alignment.

### Calculation of the eigenvalues

Calculation of the eigenvalues is done as described in (Waddell and Steel 1997). We compute, for each pair of terminal sequences, the observed divergence matrix **F**. We then compute **F**^#^, *ie*, the symmetrized form of **F**, and take the eigenvalues of **F**^#^.

### Evaluation of the alignments

After the simulations, the terminal sequences of each of the 100 datasets are aligned using ClustalW with default parameters. The quality of each inferred alignment is then evaluated by comparing it to the corresponding correct reference alignment, *ie*, we use the column score (*CS*) implemented in the BaliScore program (Thompson et al. 1999): *CS* = ∑*_i_^M^C_i_*/*M*, where *M* is the number of columns in the reference alignment and *C_i_* = 1 for a column with all bases correctly aligned, otherwise *C_i_* = 0.

## Discussion

The results of the simulations are presented in tables 1–4. Three rate matrix combinations (S1 to S3) have been considered (see above), each with four possible tree lengths (T0 to T3). We performed the simulations with 200 and 1000 base-long sequences without implementation of the insertion/deletion process. The 1000 base long sequences were analyzed as is and after extracting a substring of 200 bases (from base 200 to base 400). We also performed simulations on 200 base-long sequences with implementation of the insertion/deletion process. Table 1 shows the frequencies of negative eigenvalues inferred for each set of conditions. We also evaluated the quality (table 2) of the alignments among simulated sequences using the CS score and the frequency of wrong alignments. Finally, the percentage of observed invariant columns in the reference multiple alignments and the mean pairwise divergences among tip sequences are shown in table 3 and 4, respectively.

### Simulations without indels

The two parameters (*i*) “length of the tree” (increasing from left to right, *ie*, from T0 to T3, in all tables) and (*ii*) “difference between the two **R** matrices” (increasing from top to bottom, *ie,* from S1 to S3 in all tables) have variable impacts on the probability of observing negative eigenvalues (table 1), and/or on the accuracy of alignments (table 2), and/or on the level of divergence among sequences (tables 3 and 4). For sequences simulated on the shortest tree (T0), all inferred alignments are correct (table 2) and are characterized by an average of 44% of columns with identical sites (table 3) and an average of 31.7% different sites between pairwise terminal sequences. As shown in table 2, simulation on longer trees (T1–T3), yield sequences that can be easily aligned (as shown by the low frequency of wrong alignments and high CS scores). One notable exception is the combination of settings T3/S3, under which alignments are essentially unreliable (table 2) and characterized by an average of 61% pairwise sequence divergence (table 4).

Although the probability of observing negative eigenvalues follows a general trend similar to that of alignment inaccuracy (*ie,* increased frequency of negative eigenvalues with increasing tree length and increasing difference between **R** matrices), the problem of negative eigenvalues is more quickly acute. Indeed, with 200 nucleotide-long sequences, the mean frequency of observing at least one negative eigenvalue reaches an average of 64–77%, 89–98%, and 100% for T1, T2, and T3, respectively (table 1). In other words, although alignment inference can be excellent (eg, under T1 or T2; table 2), many pairwise comparisons can lead to negative eigenvalues (table 1). The situation is only slightly less dramatic for 1000 nucleotide-long sequences: the mean frequency of observing a negative eigenvalue reaches an average of 11–18%, 68–70%, and 99–100% for T1, T2, and T3, respectively (table 1).

In an attempt to characterize the delayed appearance of negative eigenvalues for longer sequences, we demonstrate in the Appendix that (*i*) for a two state system, negative eigenvalues appear when 25% of the sites differ, irrespective of the sequence length, and (*ii*) for a four state system, the frequency of negative eigenvalues decreases with sequence length (for a fixed level of pairwise divergence) and increases with time divergence and/or substitution rate (for a fixed sequence length). These relations are illustrated in table 1: the closest pairs of sequences (*seq3 vs. seq4* and *seq5 vs. seq6* in tree T1; *seq3 vs. seq4* in tree T3) yield the lowest frequency of negative eigenvalues. Similarly, in tree T2, the frequency of negative eigenvalues for 200 base-long sequences increases from 15–37% for sequence pairs characterized by a sum of branch lengths = 12 (*seq3 vs. seq4, seq4 vs. seq6,* and *seq5 vs. seq6*) to 59–73% for sequence pairs characterized by a sum of branch lengths = 20 (*seq3 vs. seq5*). The same trend is present but partly masked (probably because of very high divergences among sequences) for T3.

### Simulations with indels

Again, we consider here three **R** matrix combinations (S1 to S3) and four different tree lengths (T0 to T3). As we limited the maximum number of insertion/deletion events according to branch lengths (*cf* Material and Methods), the maximal number of 1- or multiple-base indels (for branches 1,2,3,4,5,6; figure 1) are: 0,0,1,1,1,1 for T0, 1,1,1,1,1,1 for T1, 1,1,3,1,3,1 for T2, and 2,1,3,4,3,5 for T3. The results compiled in table 2 indicate that, when considering that sequences experience insertion/deletion events, ClustalW yields 7–22% of incorrect alignments for T0 and at least 90% incorrect alignments for T1 and above. However, one must moderate this statistics by the observation that CS scores are reasonably high (above 0.9) for all conditions except S3/T3 (where CS = 0.123). Hence, as in the simulations that do not implement the insertion/deletion process, we observe here that a non-trivial probability of observing negative eigenvalues is reached well before genuine alignment problems arise. Finally, the relations between, on one hand, the number of negative eigenvalues and, on the other hand, time divergence and substitution rates are very similar to those observed using simulations without insertion/deletion events.

## Conclusion

Our analyses indicate that negative eigenvalues can be considered, from a practical point of view, a problem for phylogeny inference as they appear before homology assessment problems (*ie,* multiple alignment problems) arise. Indeed, our comparisons between true and inferred alignments (table 2) show that, with the exception of the combination T3/S3 (longest tree with a large shift in relative substitution rates), simulated sequences can be aligned with high accuracy (*ie,* > 90% of the sites are correctly aligned) whereas **F^#^** yields negative eigenvalues (table 1) – hence an undefined logarithmic function and inapplicable GTR model – with high frequencies, even for tree T1, *ie,* for sequences still far from saturation. The values of alignment accuracy (CS) computed here might even be underestimated as alternative algorithms to ClustalW may give more accurate alignments is some conditions (Loytynoja and Milinkovitch 2003, Gardner et al. 2005, Hickson et al. 2000, Loytynoja and Milinkovitch 2001). Furthermore, although this is rarely mentioned, real datasets can produce negative eigenvalues. For example, the comparison of human and cow cytochrome b gene third positions (Lanave et al. 1984) yields one negative eigenvalue (1, 0.423996, − 0.137941, 0.111731).

Our results under cases S2 and S3, suggest that violation of the homogeneity assumption increases the risk of observing negative eigenvalues. This point is of particular pertinence if homogeneity (classically used in most of the current implementations of the GTR model, *ie,* a single ***R*** matrix is used for the whole tree) is an invalid assumption.

Our simulations also show that the length of a DNA dataset influences the probability of occurrence of negative eigenvalues in **P**: different pairs of sequences with similar divergences may have very different probabilities of yielding negative eigenvalues, depending on the length of the sequences (*eg,* using T1, the frequency of observing at least one negative eigenvalue is 11–18% and 72–77% for 1000 and 200 character-long alignments, respectively; table 1). This result indicates that the relation between sequence length and probability of observing a negative eigenvalue is not linear such that using even longer sequences might effectively reduce the number of cases where the Waddell and Steel (1997) procedure is not applicable. A method on how modifying **P** to make the GTR model always applicable as well as a discussion on the mathematical basis for the non-linear relationship between sequence length and probability of negative eigenvalues occurrence is given in (Catanzaro et al. 2006). Note that, as we are using a four-state model of substitutions, we exclude gap-containing sites before computing eigenvalues, pairwise divergences, and other statistics.

As mentioned above, methods for computing a unique **R** matrix for the whole tree have been described. Yang and Kumar (1996), for example, suggested to average the pairwise **F^#^** matrices before calculating a global **R**, but this procedure is not immune to the above-mentioned problems (*cf* introduction). As currently implemented, optimization methods like PAUP* (Swofford 2003) or MrBayes (Ronquist and Huelsenbeck 2003) directly estimate one **R** matrix using optimization techniques under maximum likelihood without the need of calculating **log(P)**. However, inference using a model based on a single **R** for the whole tree will likely lead to underoptimization of local instantaneous relative substitution rates, whereas a model based on an **R** matrix for each tree edge will yield the best relative substitution rates for the corresponding pair of internal or tip sequences. The use of this second kind of models, eventhough computationally more intensive, could yield more accurate **P**(*t*) matrices for better maximum likelihood estimations, and also relaxes the possibly inappropriate assumption hypothesis of homogeneity along the whole tree. Such a more complex model might not provide significant gain for less divergent datasets, but should perform better for divergent ones. Note that it remains to be investigated, using AIC (Akaike 1974) or Likelihood Ratio tests (Gaut and Weir 1994), whether the CPM model should be preferentially used against other procedures robust against heterogeneous base composition across the tree (logdet model, Lockhart et al. (1994)) or allowing for variation of site-specific rates among lineages (Galtier 2001).

Note that the *one*-**R**-*per*-*edge* approach can also be applied in a maximum likelihood or Bayesian framework. In such a case, and assuming the methods implemented for parameter optimization are highly efficient, the optimal tree topology should converge towards the tree obtained by the CPM method (Catanzaro et al. 2006). The relative efficiencies of these different approaches clearly warrants further investigation.

In conclusion, we show here that datasets characterized by net transition probability matrices (**P**) with negative eigenvalues (making the GTR model or logdet correction not-applicable) can be considered a real practical issue. We also show that both variable **R** matrices across the tree and sequences length do influence the probability of observing negative eigenvalues, hence, of making the GTR model not applicable. These results suggest the need for methods (such as CPM, (Catanzaro et al. 2006)) that modify **P** for removing negative eigenvalues while still describing a biologically meaningful substitution process.

## Figures and Tables

**Table 1:** Frequency of observing at least one negative eigenvalue for each pairwise sequence comparison (*i vs j*) and across all sequence comparisons (*total*). Values are color-coded as follows: *ne* = 0, 0 < *ne* < 0.5, 0.5 ≤ *ne* < 0.8 and 0.8 ≤ *ne*.

		200				200 extracted				1000				200 with indel			

		T0	T1	T2	T3	T0	T1	T2	T3	T0	T1	T2	T3	T0	T1	T2	T3
S1	3 vs 4	0	0.05	0.30	0.62	0	0.01	0.37	0.62	0	0	0.06	0.43	0	0.03	0.31	0.65
	3 vs 5	0.02	0.33	0.69	0.78	0	0.25	0.65	0.78	0	0.07	0.43	0.74	0	0.27	0.61	0.71
	3 vs 6	0	0.26	0.53	0.75	0	0.28	0.51	0.78	0	0.06	0.21	0.72	0	0.35	0.53	0.68
	4 vs 5	0	0.32	0.49	0.79	0.01	0.23	0.53	0.76	0	0.02	0.31	0.72	0	0.29	0.47	0.83
	4 vs 6	0	0.26	0.28	0.79	0	0.19	0.34	0.76	0	0.05	0.04	0.75	0	0.27	0.23	0.74
	5 vs 6	0	0.02	0.26	0.71	0	0.04	0.29	0.70	0	0	0.01	0.62	0	0.03	0.24	0.78
	**total**	**0.02**	**0.77**	**0.96**	**1**	**0.01**	**0.64**	**0.96**	**1**	**0**	**0.17**	**0.70**	**0.99**	**0**	**0.73**	**0.93**	**1**

S2	3 vs 4	0	0.07	0.28	0.65	0	0.01	0.26	0.64	0	0	0.03	0.44	0	0.06	0.42	0.65
	3 vs 5	0	0.29	0.73	0.79	0.02	0.38	0.59	0.74	0	0.09	0.43	0.70	0	0.26	0.70	0.77
	3 vs 6	0	0.24	0.56	0.79	0	0.33	0.50	0.87	0	0.05	0.27	0.69	0	0.35	0.59	0.82
	4 vs 5	0	0.23	0.55	0.76	0	0.27	0.49	0.74	0	0.02	0.19	0.69	0.01	0.20	0.45	0.77
	4 vs 6	0	0.31	0.23	0.82	0	0.32	0.35	0.84	0	0.03	0.02	0.85	0	0.21	0.27	0.79
	5 vs 6	0	0.03	0.24	0.69	0	0.03	0.33	0.76	0	0	0.04	0.64	0	0.02	0.33	0.76
	**total**	**0**	**0.72**	**0.96**	**1**	**0.02**	**0.75**	**0.94**	**1**	**0**	**0.18**	**0.68**	**1**	**0.01**	**0.64**	**0.97**	**1**

S3	3 vs 4	0	0.04	0.32	0.67	0	0.03	0.26	0.64	0	0	0.06	0.43	0	0.06	0.35	0.69
	3 vs 5	0	0.37	0.68	0.74	0.01	0.34	0.65	0.82	0	0.05	0.48	0.68	0.01	0.24	0.66	0.77
	3 vs 6	0	0.28	0.58	0.99	0	0.29	0.62	0.99	0	0.03	0.32	0.99	0	0.24	0.61	0.98
	4 vs 5	0	0.32	0.55	0.79	0.01	0.36	0.57	0.83	0	0.03	0.23	0.83	0	0.26	0.49	0.81
	4 vs 6	0	0.31	0.26	1	0	0.25	0.22	0.98	0	0.02	0	1	0	0.22	0.23	0.98
	5 vs 6	0	0.04	0.29	0.99	0	0	0.15	0.99	0	0	0.02	0.96	0	0.02	0.25	0.96
	**total**	**0**	**0.74**	**0.98**	**1**	**0.02**	**0.70**	**0.89**	**1**	**0**	**0.11**	**0.68**	**1**	**0.01**	**0.63**	**0.97**	**1**

**Table 2:** Accuracy of sequence alignments using ClustalW (Thompson et al. 1994). Frequency (*f*) of wrong alignments, mean ( $\hat{CS}$) and standard deviation (*CS_sd_*) of the CS scores (100 simulations). Values are color-coded as follows: *f* = 0, 0 < *f* ≤ 0.9 and 0.9 < *f*.

			T0			T1			T2			T3	

		f	$\hat{CS}$	*CS_sd_*	f	$\hat{CS}$	*CS_sd_*	F	$\hat{CS}$	*CS_sd_*	f	$\hat{CS}$	*CS_sd_*
S1	200	0	1.000	0.000	0	1.000	0.000	0.02	0.999	0.006	0.02	0.999	0.005
	200ext	0	1.000	0.000	0	1.000	0.000	0.01	1.000	0.004	0.05	0.997	0.013
	1000	0	1.000	0.000	0	1.000	0.000	0	1.000	0.000	0.08	0.998	0.008
	200ID	0.17	0.985	0.011	1	0.952	0.028	0.99	0.958	0.025	1	0.928	0.033

S2	200	0	1.000	0.000	0	1.000	0.000	0	1.000	0.000	0.03	0.998	0.010
	200ext	0	1.000	0.000	0.01	0.999	0.009	0	1.000	0.000	0.04	0.998	0.010
	1000	0	1.000	0.000	0	1.000	0.000	0	1.000	0.000	0.07	0.999	0.005
	200ID	0.22	0.988	0.009	0.98	0.966	0.020	0.97	0.956	0.026	1	0.907	0.044

S3	200	0	1.000	0.000	0.1	0.991	0.031	0.05	0.996	0.019	1	0.127	0.175
	200ext	0	1.000	0.000	0.09	0.995	0.016	0.07	0.996	0.018	1	0.147	0.215
	1000	0	1.000	0.000	0.18	0.996	0.010	0.18	0.996	0.010	1	0.069	0.083
	200ID	0.07	0.974	0.016	0.9	0.969	0.033	1	0.914	0.058	1	0.123	0.160

**Table 3:** Percentage and *standard deviation* of identical columns in the multiple alignments.

		T0		T1		T2		T3	
S1	200	45	*4*	25	*3*	20	*2*	15	*2*
	200 ext	43	*4*	26	*3*	20	*3*	15	*2*
	1000	44	*2*	26	*1*	20	*1*	15	*1*

S2	200	46	*4*	26	*3*	19	*3*	15	*3*
	200 ext	44	*3*	26	*3*	20	*3*	15	*2*
	1000	44	*2*	26	*2*	20	*1*	15	*1*

S3	200	44	*4*	23	*3*	18	*2*	13	*2*
	200 ext	43	*3*	24	*3*	18	*3*	13	*3*
	1000	43	*2*	24	*1*	18	*1*	13	*1*

	average	44		25		19		14	

**Table 4:** Percentage and *standard deviation* of mean pairwise divergence among tip sequences.

		T0		T1		T2		T3	
S1	200	31	*5*	43	*5*	46	*4*	51	*4*
	200 ext	32	*5*	42	*5*	47	*4*	51	*4*
	1000	31	*4*	43	*4*	47	*3*	51	*2*

S2	200	30	*5*	43	*5*	47	*4*	52	*4*
	200 ext	31	*5*	43	*5*	47	*4*	52	*4*
	1000	31	*4*	43	*4*	47	*2*	52	*2*

S3	200	32	*5*	47	*6*	50	*5*	61	*11*
	200 ext	33	*5*	46	*6*	50	*5*	61	*11*
	1000	33	*4*	46	*5*	51	*4*	61	*11*

	average	31.7		43.9		47.9		54.6	

## Footnotes

1 When applying the Waddell and Steel (1997) procedure on AACGTGGCCAAAT, ATCGTCGTTAACC and AATTTCGTCACAA, the pairs of sequences (1,2) and (2,3) exhibit negative eigenvalues even when averaging the **F**# matrices.

## Appendix

### On the relationship between sequence length and occurrence of negative eigenvalues

#### Theorem

Let’s consider two sequences, s_1_ and s_2_ ∈ ∑ = {X, Y}, of two-state characters where ∑ = {X, Y} is the set of all possible DNA sequences having length l. When the number of differences between s_1_ and s_2_ is greater than 0.25*l, then **F**^#^ will be characterized by at least one negative eigenvalue.

##### Proof

Let’s consider the divergence matrix (Waddell and Steel 1997) **F** of *s*_1_ and *s*_2_

(7) $$F=\left(\begin{array}{cc} a & b\\ c & d \end{array}\right)$$

and its symmetrized form

(8) $$F^{\#}=\left(\begin{array}{cc} a & e\\ e & d \end{array}\right)$$

where e = (*b* + *c*)/2. Since

(9) a+b+c+d=l

that is

(10) a+d+2e=l

then **F**^#^ can be rewritten as

(11) $$F^{\#}=\left(\begin{array}{cc} a & e\\ e & l-a-2e \end{array}\right)$$

The symmetric matrix **F**^#^ is positive definite (*ie,* characterized by strictly positive eigenvalues) if and only if the Sylvester’s criterion (Press et al. 2002) is satisfied:

(12) a>0

(13) $$a\;(l-a-2e)>e^{2}$$

(12) imposes that the number of equal characters among *s*_1_ and *s*_2_ must be a positive number; while (13) can be modified as follows:

(14) $${\left(e+a\right)}^{2}<al$$

that is (by excluding negative solutions because they have no physical meaning)

(15) $$e<\sqrt{al-a}$$

By considering *l* assigned, the maximum number of different characters between *s*_1_ and *s*_2_ for which **F**^#^ is still characterized by positive eigenvalues can be obtained by deriving (15) with respect to *a:*

(16) $$\frac{\partial }{\partial a}e_{|l=const}=-1+\frac{l}{2\sqrt{al}}=0$$

obtaining so

(17) a=l/14

By substituting (17) in (15) we find that *l*/4 is the maximum value that *e* (the number of differences between *s*_1_ and *s*_2_) can take such that **F**^#^ is still characterized by positive eigenvalues. The relation *e* < *l*/4 is a necessary, although not sufficient condition for **F**^#^ to be characterized by positive eigen value. Q.E.D.

When analyzing four state-character sequences, the matrix **F** becomes:

(18) $$F=\left(\begin{array}{cccc} f_{11} & f_{12} & f_{13} & f_{14}\\ f_{21} & f_{22} & f_{23} & f_{24}\\ f_{31} & f_{32} & f_{33} & f_{34}\\ f_{41} & f_{42} & f_{43} & f_{44} \end{array}\right)$$

By calling **F**^#^ the symmetrized form of the divergence matrix **F**, the maximum number of differences between two sequences of length *l* such that **F**^#^ is characterized by strictly positive eigenvalues can be obtained by solving the following non-linear optimization problem (Bertsekas 1999):

(19) $$maximize\sum_{i^{1}j}f_{ij}^{\#}$$

(20) $$s.t.\;\sum_{i=1\ldots 4}\sum_{j=1\ldots 4}f_{ij}=l$$

(21) $$Det(F_{k}^{\#})>0,\;\;\;k=1\ldots 4$$

where **F***_k_*^#^ is the *k*-order minor of **F**^#^. Constraint (20) imposes that the sum of the *f**_ij_* must be equal to the length of the sequences; constraint (21) imposes the Sylvester’s Criterion (Catanzaro et al. 2006). The two state-characters condition is nested in the above formulation and represents an underestimation of the maximum number of differences. In other words, when analyzing a pair of four-state character sequences, if the number of differences between the two sequences is smaller than *l*/4 then the matrix **F**^#^ cannot be characterized by negative eigenvalues.
